# Report of Twenty-Seven Operations for Cataract

**Published:** 1874-09

**Authors:** A. W. Calhoun

**Affiliations:** Atlanta, Ga.


### REPORT OF TWENTY-SEVEN OPERATIONS FOR CATARACT,
### With Remarks upon Some of the Modes of Operating.

By A. W. CALHOUN, M.D., Atlanta, Ga.

That something is needed to perfect the various operations for the relief of cataract, is evidenced by the multitude of suggestions coming from ophthalmologists in every country. Such a variety of opinions, arising in every direction and from the best of sources, indicates a restlessness amongst this class of surgeons, brought about, doubtless, by a need of some positive plan upon which we can always base a mode of operating. As a consequence of this, new operations are being constantly developed, exist but a brief period, and then cease to be heard of, except as mere matter of history.

But from all this, some good is being derived; for, while many of the suggested operations, as a whole, may be discarded, some single ideas are often retained and adopted as modifications in the more general modes of operating, and are thus gradually advancing us to that end we so much desire, viz: an operation which is complete in all its details, and affording the best possible chances to the patient.

The operation known as Graefe's is, by common consent, acknowledged to be the one best suited to certain forms of cataract; in other words, to the large majority of cases; but in reading the reports of cases, and in hearing surgeons speak of their operations, we are struck with the fact that few, if any, adhere to Graefe's original idea in full, but that very nearly all hold to it simply as a ground work, each deducting certain minutiæ, and adding modifications peculiar to himself.

Of the twenty-seven operations I have comparatively recently made, the extractions were not all performed after Graefe's plan; but keeping the main principles in view, I have also added to or deducted certain things which appeared to me to be of use in the completion of the operation, and which in the main have served me quite well.

Of the above number, six extractions were performed with Beer's knife, and by making a large flap upwards; the other steps in the operation being just the same as in the linear extraction. The only difference, then, between the latter, or Graefe's mode, and that practiced in the above cases, is that the broad knife was used, enabling me to make a larger wound for the exit of the lens, and also, at that time, being more readily used by myself than the long narrow knife used at present in performing the linear extraction.

A few years ago, some of the most prominent oculists in this country, and even now several of the English surgeons, particularly at Moorefield's Ophthalmic Hospital in London, make use of this kind of a knife (Beer's or Sichel's) in preference to any other, it being more easily handled than the long knife of Graefe, and the extent of the wound being more accurately regulated. Critchett, one of the most celebrated of London oculists, brings it into constant use.

The recoveries of these six cases thus operated upon were perfect in every particular; the vision being, in each case, brought up to as near a normal standard as is usually the case after such an operation. The ages of five of them ranged from sixty to eighty-two years, but in each instance the recovery was not in the least hindered, although one or two of them were in feeble health. The sixth case was twenty-six years old, and had congenital lamellar cataract. Upon her the same plan of operating was followed, she not being able or willing to undergo the more tedious operation of discission or laceration; and the result was that she now superintends her own business—that of farming—and for a time even taught successfully a small school.

In addition to this, there were twelve cases upon whom the linear extraction was performed, as near as practicable, in entire conformity with the idea of Graefe, using the narrow and slender knife now almost altogether used in making this operation. Of these, eight made rapid and good recoveries; vision being also restored to more than half the normal degree in each instance, and in several, to as near a natural state as could be hoped for. Amongst the remaining four, two had severe iritis to follow the operation, interfering materially for the time being with the complete success that was promised for several days, by the formation of secondary cataract, an exudative membrane produced by the inflammatory process, and filling up the pupil to greater or less extent. One of these two has since regained tolerable vision, by the almost complete absorption of this membrane. The other requires only a simple needle operation to rupture the membrane, and thus permit of good vision here also. In one case, upon which two other operations were performed, the double cataract was the result of severe irido-cyclitis in each eye, which existed in a chronic state at the time of the operation. It was necessary to make extensive iridectomies to relieve the complete posterior synechiæ, which were the causes of the violent pain and the continuation of the inflammation, and at the same time both lenses were extracted. Although comparative success was promised (the patient being able to count fingers immediately after the operation), the old disease (irido-cyclitis) sprang up acutely after a few days, and continued, notwithstanding the most active treatment, till the pupils became totally occluded by a thick membrane, completely cutting off all entrance of light into the interior of the eye. The prognosis of this case was so unfavorable that it was not deemed advisable to interfere further.

So, then, to sum up the result of the twelve linear extractions, eight came through with no undue inflammation and good vision; one had iritis, and as a consequence, a membrane, filling the pupillary space, but it ultimately disappeared by absorption, leaving still fair sight; another suffered similarly (from iritis), and the thickness of the membrane will require the use of the needle to break it up, when, in all probability, the vision will be as perfect as though no secondary inflammation had arisen to even temporarily obstruct it. The other case, upon whom the *two* operations were made, had an acute attack of the chronic affection (irido-cyclitis), destroying the slight vision that was gained immediately after the operation.

It is well to remark, just here, that in this last case the operations were made not so much for the relief of the cataract (though at the time it was hoped that the extraction would result in some good,) but more especially to relieve the synechia posterior and the chronically inflamed condition of the iris, and the consequent pain. The last, I hope and believe I have succeeded in fully overcoming.

Upon children, nine operations were made, and, as usual in young persons, the character of the cataract was soft, requiring the operation known as discission. In a few of the cases it was necessary to repeat the operation several times before complete absorption had taken place; while in others a few strokes of the needle seemed to dissipate the opacity, the lens substance having become quite fluid (morgagnian cataract), needing only a rupture of the anterior capsule to allow it to flow out and become mixed with the aqueous humour. In each case the cataract was congenital; and their ages, at the time of the operation, were from five to thirteen years. As is well known, operations for complete congenital cataract are rarely *markedly* successful, from the simple fact that the sensibility of the retina is nearly always obtunded; that is, amblyopia to a greater or less degree exists, and increases more and more, the longer the cataract is allowed to remain. On this account it is advisable to operate upon children with congenital cataract as soon as their age and condition will allow it, so that the retina may be brought into early exercise, whereby alone it can be restored to its natural function.

With the exception of one case, operated upon some time since, and still another now under treatment, all were materially benefitted, and a few restored to a remarkable and unexpected degree of vision. Not a single case suffered from undue inflammation, but each grew rapidly well, being usually ready, when necessary, to have the operation repeated after two or three weeks.

There are three, aged respectively five, nine and thirteen years, who, up to the time of their discharge, had so far improved as to be able to go about, even in strange places, unaided and unattended—an impossibility prior to the operation. The flattering results, in two of these cases, I think I can in great measure attribute to the use of strychnine, beginning with the fortieth of a grain (hypodermically) daily, and increasing the dose gradually to the fourth or fifth of a grain. The good effect of this medicine in atrophic conditions of the optic nerve, has long since been well established, and the results of its use in these two, as well other cases, have been very satisfactory indeed.

To recapitulate, of eighteen extractions, all were successful except two cases. One of these now needs only a rupture of the pupillary membrane, by means of the needle, to be restored to good vision. The other was originally a most unfavorable case, (chronic irido-cyclitis, with secondary cataract,) and it was at first only intended to make an iridectomy for the relief of pain and other troublesome symptoms; but it was afterwards determined to remove the diseased lens at the same time. The pain was relieved, but the extraction was of only temporary benefit.

Of the nine discissions in children, with one exception all were restored to more or less vision. The one mentioned as an exception was benefited, but not to any great degree. It was a little girl of thirteen years, with ill-developed and constantly moving (nystagmus) balls and congenital cataract in both eyes. Another, upon whom I recently operated, is still under treatment, and will probably need a repetition of the operation once or twice more before complete absorption takes place.

The fact that a child with congenital cataract has tolerable perception of light, or can distinguish between the principal colors when held before it, is not always an evidence that success will follow an operation and vision be perfected. I have often seen just such cases, and have myself had one or two, where one unaccustomed to making these operations would naturally imagine that a simple removal of the lens would be productive of good sight. Often, however, is vision not one particle benefited, the little patient seeing just as much, but not a whit more, after the absorption of the lens as before. The amblyopia, or impairment of the more sensitive elements of the retina, perhaps from non-use, has existed so long that the removal of the probably original trouble (the opacity of the lens) does not increase the vision. It is barely possible that in such cases, long continued active exercise of the retina, and the use of some powerful nervous stimulant, as strychnine, will ultimately restore more or less vision.

Amongst oculists, one of the greatest fears in all operations having the extraction of the lens in view, is the loss of vitreous humour, and in every, so-called, improved operation that is paraded before the profession, the promise to more effectually prevent such an escape of vitreous, is indulged in as an evidence of superiority in the new mode.

My experience in witnessing and following up an immense number of extractions in most of the large hospitals of Europe, and in this country, and my own personal experience, lead me to believe that this fear is greatly exaggerated. Indeed, Hasner, Professor of Ophtalmology in the University of Prague, makes it a point after each extraction to rupture the posterior lenticular capsule, and allow the vitreous humour to flow into the anterior chamber, whereby more or less is invariably lost by being washed out through the corneo-sclerotic wound at the moment of rupture. His object is, by the bulging forward of the vitreous, to make a rent through the posterior capsule, and whatever remnants of cortical substance of the lens may be remaining behind, thereby preserving a clear opening, even though secondary cataract should supervene.

Pagenstecher, Professor in Wiesbaden, makes a very successful operation for extraction of cataract, peculiar to himself, in which, in a very large majority of cases, a large quantity of the vitreous escapes. Nothing is done to prevent it, more than that ordinary caution is exercised. Not the least harm ever results from such an accident, (as it is usually so considered) and the various steps of the operation are proceeded with in the usual manner, as though nothing had gone wrong.

It is true that an escape of vitreous humour during an operation is not desired, for it interferes very much with the progress of the operation, unless it has already been brought nearly to an end, as is the case most frequently.

Oftentimes have I seen the fourth or fifth, and even more, of all the vitreous escape without hindering, in the least, the perfect healing of the wound, and with as good vision as could possibly have otherwise been obtained. Loss of vitreous previous to the exit of the lens, is indeed a serious complication; for each successive attempt to deliver the lens only tends to increase the outflowing of vitreous humour. Such an accident is, however, of but rare occurrence. Most frequently, the vitreous begins to rush out after the removal of the lens, it being then deprived of its support from the front, when a large quantity can be lost not only without any hinderance to the healing process and good vision, but I am inclined to the belief that it is an actual benefit, inasmuch as the tendency to serious inflammation is apparently much reduced after such an escape, provided the iris may have been freed from any pressure or traction, from being caught in the corneo-sclerotic wound as the vitreous flowed out, and washed the iris along with it.

The operation of *iridotomy*, as performed by De Wecker, of Paris, mostly for the relief of secondary membranous cataract, can not unfrequently also be put to good use in certain cases as an auxiliary to the ordinary mode of extraction. For example, in those cases where cataract is secondary—that is, more particularly the result of a chronic inflammation existing in the iris or iris and ciliary bodies—the iridectomy, or this, with the bruising of the iris incident to the passage of the lens, has a tendency to start the chronic inflammation afresh; as a consequence of which the iris substance swells and the edges of the iridectomy approach each other, oftentimes meeting and adhering, causing complete obliteration of the pupil, and the iris to appear as if no pupil had ever existed. Under such circumstances the occlusion is not altogether caused by a membrane simply filling up the pupillary space, but by the edges both of the natural and artificial pupils being drawn together and thus uniting. Now it has occurred to me to be of benefit, and I have put it into execution with success in a few cases, to combine the *iridotomy* in the lower portion of the iris, with the *iridectomy* in the upper portion, in those cases of extraction where, from long continued iritis or irido-cyclitis, the tendency is for the pupil to become entirely obliterated by the approach and union of the cut edges. By such a procedure, the iris is divided more or less into two halves, and the circular muscular fibres (the sphincter) are severed at two opposite points, thereby giving complete rest to each portion. Even though acute inflammation should then spring up out of the chronic, the contraction of the iris tissue would be inclined rather to draw the two halves away from each other; so that, notwithstanding the exudative deposit, enough clear space might be left between the cut edges of the iris to allow of tolerable vision.

Of course, in ordinary uncomplicated cataract—in short, in any except such cases as just mentioned—an *iridotomy* is not to be thought of, as it would not only be unnecessary, but might be of injury; at all events of great disadvantage. In simple cataract, the least done to the iris the better.

